# When Seborrheic Keratosis Is Wearing a Mask

**DOI:** 10.5826/dpc.1002a38

**Published:** 2020-04-20

**Authors:** Cristina Sousa, Sebastian Podlipnik, Susana Puig, Josep Malvehy

**Affiliations:** 1 Dermatology Department, Hospital Clínic, Barcelona, Spain; 2 Dermatology Department, Centro Hospitalar de Vila Nova de Gaia/Espinho, Vila Nova de Gaia, Portugal

**Keywords:** seborrheic keratosis, fusion tumor, dermoscopy, dermatology challenge

## Case Presentation

**Figure 1:**
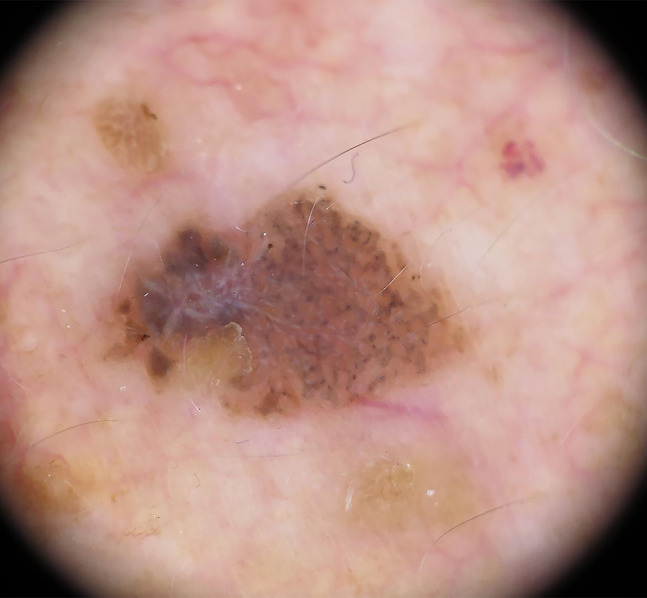
Dermoscopy image of a cutaneous lesion presenting with a multicomponent pattern, with 4 colors, a globular atypical pattern, pseudonetwork, and asymmetrical globules.

A male patient, 72 years old, had a history of melanoma (AJCC stage IB) located on the left leg. During a regular follow-up visit, we observed an asymmetric, brown, blue-gray plaque with 1.2 cm maximum diameter, sharply delimited on the right supraclavicular region. On dermoscopy, the lesion presented a multicomponent pattern, with 4 colors (brown, gray, blue, and white), a globular atypical pattern, pseudonetwork, and asymmetrical globules. In the left area of the image, it was possible to see a homogenous brown area, with overlapping gray and white shiny structures and ovoid nests which led to the suspicion of a collision tumor of seborrheic keratosis and basal cell carcinoma or melanoma, mainly because of these two distinctive patterns on the same area (Figure 1). The lesion was excised, and the histology was compatible with a seborrheic keratosis.

## Teaching Point

At times, the clinical and dermoscopic diagnosis of seborrheic keratosis is challenging because it can mimic other conditions including basal cell carcinomas or melanomas [1,2].
